# Supplementary material for: Conditional chemoconnectomics (cCCTomics) as a strategy for efficient and conditional targeting of chemical transmission
Source: eLife. 2024 Apr 30;12:RP91927. doi: 10.7554/eLife.91927 (PMC11060718; doi:10.7554/eLife.91927)
Supplement: Figure 3—source data 3. [file elife-91927-fig3-data3.zip › Figure 3 source data3 part 1.zip!/Figure 3-Source data 3-part1/1175-9-CRISPResso_Report_rtnd62ko.zip!/CRISPResso_on_1175_11/2a.Nucleotide_percentage_quilt.pdf]

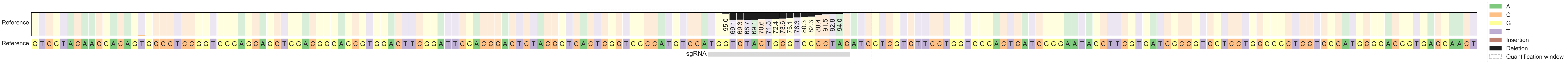
Reference
95.0 69.1 69.3 68.7 69.1 70.6 71.5 72.4 73.6 75.1 78.3 80.3 82.3 88.4 91.5 92.8 94.0
Reference
GTCGTACAACGACAGTGCCCTCCGGTGGGAGCAGCTGGACGGGAGCGTGGACTTCGGATTCGACCCACTCTACCGTCACTCGCTGGCCATGTCCATGGTCTACTGCGTGGCCTACATCGTCGTCTTCCTGGTGGGACTCATCGGGAATAGCTTCGTGATCGCCGTCGTCCTGCGGGCTCCTCGCATGCGGACGGTGACGAACT
sgRNA
A
C
G
T
Insertion
Deletion
Quantification window
